# 

**Published:** 1911-06-03

**Authors:** 


### BOOKS RECEIVED.

J. Bale, Sons, and Danielsson, Ltd.

"History of Infant Feeding from Elizabethan Times." By David Forsyth, M.D.

"Life History, Function, and Inflammation of the Appendix.' By E. M. Corner.

W. B. Saunders Company.

"State Registration for Nurses." By Louie Croft Boyd, R.N.

T. G. and E. G. Jack.

"The Woman's Book." By Florence B. Jack.

Surgery Publishing Company.

"One Thousand Surgical Suggestions." By Walter M. Brickner, B.S., M.D.

### MEDICAL APPOINTMENTS, ETC.

Dr. Hector Charles Cameron, M.D. Camb., has been appointed Assistant Physician to Guy's Hospital.

Dr. Thomas Buzzard, M.D., F.R.C.P., has been appointed Governor of Westminster Hospital Medical School.

The following appointments have been made on the honorary medical staff at Salford Royal Hospital: To be honorary surgeons, Garnett Wright, Esq., M.B., Ch.B., Edin., F.R.C.S. Eng.; Robert Ollerenshaw, Esq., M.D., Ch.B. Vict., F.R.C.S. Eng., L.R.C.P. Lond. Honorary assistant physicians, F. Edward Tylecote, Esq., M.D., Ch.B., D.P.H. Vict., M.R.C.P. Lond.; C. Christopher Heywood, Esq., M.A., M.B., B.C. Cantab., M.R.C.P. Lond., M.R.C.S. Eng. Honorary assistant surgeons, James Barlow Macalpine, Esq., M.B., Ch.B. Vict., L.R.C.P. Lond., F.R.C.S. Eng.; J. Philip Buckley, Esq., M.A., B.C. Cantab., M.B., B.S. Lond., F.R.C.S. Eng.

### BUILDINGS CONTEMPLATED AND IN PROGRESS.

Bude.—For the erection of an isolation hospital, the Urban District Council have accepted a tender of £375.

Buxton.—A site for a cottage hospital has been given by the Duke of Devonshire, and the building works will shortly be commenced.

Cheadle (Staffs).—Alterations and additions are contemplated to the isolation hospital. Particulars may be had from the surveyor to the Cheadle Rural District Council, Mr. W. P. Inskip.

Haywards Heath.—For the erection of an Edward Memorial Cottage Hospital, Messrs. Wheeler and Godman, of Horsham, are architects; a tender of close on £3,000 has been accepted for the works.

Keighley.—The Cottage Hospital Committee have decided to extend the buildings to provide more male accommodation (seven beds) and staff rooms, at a cost of about £500.

Canterbury.—Mr. F. H. Doré, of Watling Street, Canterbury, is architect for the proposed extension of the Cottage Homes.

Hartismore.—The Hartismore Board of Guardians have instructed their Site Committee to proceed with the purchase of land and to obtain plans for a new infirmary.

Kelso.—Mr. J. P. Alison, F.R.I.B.A., of Hawick, is architect for the restoration of Kelso Poorhouse.

Lanchester.—Mr. G. T. Wilson, Licentiate R.I.B.A., is carrying out certain renovation works at the Workhouse and Cottage Home Buildings for the Guardians of the Union.

London.—The Metropolitan Asylums Board have appointed Mr. T. W. Aldwinckle as architect in connection with the provision of additional isolation accommodation at the North-Western Hospital, at a commission of 5 per cent. on the total cost of the works.

### GIFTS.

Miss Marianne Wilkin, of Bournemouth, bequeathed £1,000 to the Royal Victoria Hospital, Bournemouth.

Mr. William Coleman, of Dover, has bequeathed £1,000 to the Royal Victoria Hospital at Dover, for the endowment fund.

The late V. K. Armitage, Esq., has bequeathed to the Salford Royal Hospital, for general purposes, a legacy of £1,000, free of duty.

Mrs. Julia Giradet, Bournemouth, has bequeathed £100 each to Barrington's Hospital, Limerick, the Victoria Home for Cripples, Bournemouth, and to the Royal Boscombe Hospital.

Mr. John Edmond Thomas, of Harrogate, has left £100 each to the Harrogate Infirmary, the Rawson Convalescent Home, Harrogate, and the Yorkshire Home for Incurables, Harrogate.

Mr. John Snow MacKinnon, of Fairfield, Liverpool, has left the ultimate remainder of his estate—as to £1,000 to endow a bed in the hospital in Killigrew Road, Falmouth; and the ultimate residue, which it appears will amount to about £6,000, to Dr. Stephenson's Children's Homes and Orphanages, Victoria Park, N.E.

Mr. George Groves, of Oddington, Glos., farmer, who left estate of the gross value of £10,569, bequeathed to each tenant of his cottages of two years' occupation half a year's rent; and £100 each to the Cottage Hospital, Moreton-in-the-Marsh, the Cottage Hospital, Bourton-on-the-Water, and the Radcliffe Infirmary, Oxford.

### JOTTINGS.

The Credito Italiano of Rome has sent £100 to the Italian Hospital in Queen Square, W.C.

In order to commemorate the Coronation the London Orphan Asylum, Watford, has decided to admit all the fifty-eight candidates now upon the list. No midsummer election, therefore, will be held. The King, who is patron of the Asylum, has expressed his "appreciation" of this decision.

A sum of £4,625 has been subscribed towards the Surrey Memorial to King Edward. The fund has closed. It is hoped to raise £5,500 to provide an endowment of £5,000 for the Surrey Convalescent Home for Children and a replica of Sir Luke Fildes's State portrait of the late King, which is to be hung in the County Hall at Kingston.

### APPOINTMENTS VACANT.

Full particulars of these vacancies can be obtained on reference to our advertisement columns:

Beckett Hospital, Barnsley.—Second House Surgeon.
Charing Cross Hospital.—Clinical Pathologist and Bacteriologist.
Coventry and Warwickshire Hospital.—Junior House Surgeon.
Leicester Infirmary.—Senior House Surgeon, House Surgeon, and Assistant House Physician.
Royal Albert Hospital, Devonport.—Assistant House Surgeon.
Wolverhampton and Staffordshire General Hospital.—House Surgeon.
Cardiff Union.—Assistant Medical Officer.
Crichton Royal Institution, Dumfries.—Junior Assistant Physician.
Kent and Canterbury Hospital.—House Physician.
Manchester Royal Infirmary.—House Physicians and House Surgeons.
Manchester Royal Infirmary (Cheadle Convalescent Hospital).—Assistant Medical Officer.
Royal Surrey County Hospital, Guildford.—Assistant House Surgeon.
The Prince of Wales's General Hospital, Tottenham, N.—Honorary Surgeon.

